# Effects of mindful physical activity on perceived exercise exertion and other physiological and psychological responses: results from a within-subjects, counter-balanced study

**DOI:** 10.3389/fpsyg.2023.1285315

**Published:** 2023-11-10

**Authors:** Payton Solk, Lisa A. Auster-Gussman, Emily Torre, Whitney A. Welch, Karly Murphy, Julia Starikovsky, Jean M. Reading, David E. Victorson, Siobhan M. Phillips

**Affiliations:** ^1^Department of Preventive Medicine, Northwestern University Feinberg School of Medicine, Chicago, IL, United States; ^2^Department of Medical Social Sciences, Northwestern University Feinberg School of Medicine, Chicago, IL, United States

**Keywords:** moderate to vigorous physical activity, guided meditation, perceived exertion, health behavior, mindful physical activity

## Abstract

**Background:**

Most adults are insufficiently active. Mindfulness training may increase moderate to vigorous physical activity (MVPA) adoption and adherence. However, physiological and psychological factors underlying these effects are not well understood. This study examined the effects of an acute bout of MVPA, mindfulness training, and combined MVPA and mindfulness training on physiological and psychological outcomes.

**Methods:**

Healthy adults (*N* = 29, M_age_ = 28.6) completed 20-min counterbalanced conditions: (a) mindfulness training (MIND); (b) moderate intensity walking (PA), and (c) moderate intensity walking while listening to MVPA-specific guided mindfulness training (PAMIND). Heart rate (HR), Rating of Perceived Exertion (RPE), Feeling Scale (FS) and Blood Pressure (BP) were measured at rest, at regular intervals during each condition, and post-condition. Mindfulness, state anxiety, and self-efficacy were assessed pre- and post-condition.

**Results:**

Average and peak HR, systolic BP (SBP), and RPE were significantly higher, and average and peak FS were significantly lower during the PA and PAMIND conditions compared to MIND (*p* < 0.001). Average RPE was significantly higher for PA compared to PAMIND (*p* < 0.001). Heart rate, feeling scale, body and mental events mindfulness, and self-efficacy for walking increased from pre to post (all *p*’s < 0.001) for all conditions. Time by condition interactions were significant for change in heart rate, mental events mindfulness, and state anxiety from pre- to post-condition.

**Conclusion:**

The physiological response to MVPA and PAMIND were similar. However, RPE was rated lower in the PAMIND condition, which could have implications for MVPA adoption and maintenance. Future work should further explore RPE combining MVPA and mindfulness training.

## 1. Introduction

Moderate to vigorous physical activity (MVPA) is associated with reduced risk of numerous chronic conditions [i.e., diabetes, cardiovascular disease and certain cancers (Warburton et al., 2006; U.S. Department of Health and Human Services, 2018), depression, stress, and anxiety (Ensari et al., 2015; Mikkelsen et al., 2017)]. Moderate intensity activity (e.g., walking briskly, dancing, doubles tennis, slow swimming) is typically defined as any activity that requires three to six times as much energy per minute as being at rest [i.e., 3.0–6.0 metabolic equivalents (METS)] or elevates heart rate to 40 to 60% of heart rate reserve or 65%–76% of maximum heart rate (MacIntosh et al., 2021). Vigorous intensity activities (e.g., jogging, running, strenuous fitness class, fast swimming) burn more than 6 METs or elevates the heart rate to 60%–84% of heart rate reserve or 77%–93% of maximum heart rate (MacIntosh et al., 2021). Despite the benefits of MVPA, physical inactivity is prevalent globally, and less than half of the global adult population meet the World Health Organization (WHO) physical activity guidelines of 150 min of MVPA per week (World Health Organization, 2019; Bull et al., 2020; Cunningham et al., 2020). Physical activity research has been conducted widely and has focused on various key areas including health benefits of physical activity (Penedo and Dahn, 2005; Warburton et al., 2006), sedentary behavior reduction (Rezende et al., 2014; Park et al., 2020), feasibility, acceptability and efficacy of physical activity interventions (Howlett et al., 2018), technology and physical activity (Hakala et al., 2017; Brickwood et al., 2019), motivation and behavior change factors of physical activity participation (Teixeira et al., 2012; French et al., 2014; Young et al., 2014), and environmental and policy factors associated with physical activity (Humpel et al., 2002; Smith et al., 2017). Despite the evidence supporting MVPA participation and large scale public health physical activity promotion efforts, there has been little change to the rate of adherence to aerobic physical activity recommendations (Ding, 2018). Given the lack of MVPA uptake, it is essential to identify novel and effective strategies to promote MVPA adoption and maintenance.

Although various factors might contribute to low adherence to MVPA guidelines, one potential modifiable, low cost, and scalable target for remediation is promoting mindfulness practice (Schneider et al., 2019; Yang and Conroy, 2020). Mindfulness is characterized by paying attention to one’s present moment experiences with curiosity, openness, and awareness of thoughts, feelings, and physical sensations (Bishop et al., 2004; Schneider et al., 2019). Mindfulness-based interventions are ubiquitous in healthcare and have demonstrated effectiveness in improving emotion regulation and decreasing symptoms of depression, anxiety, and a host of physical symptoms and side effects associated with chronic disease and its treatment (Victorson et al., 2015). Emerging data indicate mindfulness training that occurs independently from physical activity may increase adoption and maintenance of MVPA participation (Ulmer et al., 2010; Loucks et al., 2015; Ruffault et al., 2017; Meyer et al., 2018). A recent meta-analysis found moderate effects for increasing physical activity following mindfulness training in adults with overweight/obesity (Ruffault et al., 2017). Further, increased mindfulness is associated with higher likelihood to follow through on MVPA intentions (Chatzisarantis and Hagger, 2007) and maintain an exercise program (Ulmer et al., 2010). Thus, engagement in mindfulness training may be an effective, scalable strategy for increasing MVPA.

Although existing literature supports the association between mindfulness and MVPA participation, less is known about factors that explain this relation. Many physiological and psychological factors have been hypothesized to explain the underlying potential relationship between mindfulness training and physical activity. Firstly, mindfulness may enhance MVPA participation through mitigation of uncomfortable physiological reactions, downregulation of negative affect [e.g., displeasure, discomfort (Lind et al., 2009; Cox et al., 2018)], increased positive experiences during MVPA and enhancement of behavioral and psychological factors [e.g., self-regulation, motivation, habit (Schneider et al., 2019)]. Further, physiological stress, associated discomfort, and perceived exertion may contribute to negative affective associations with MVPA, (Lind et al., 2009) which have been associated with reduced MVPA participation (Cox et al., 2018). Mindfulness is associated with decreased physiological stress markers including slower respiratory rate, decreased heart rate, and decreased systolic and diastolic blood pressure (Ditto et al., 2006; Kingston et al., 2007; Delgado et al., 2010; Pascoe et al., 2017; Gamaiunova et al., 2022). Mindful awareness may enhance regulation of physiological and affective responses and lessen the reactivity to discomfort experienced during MVPA (Kabat-Zinn, 1982; Arch and Craske, 2006; Chambers et al., 2009; Schneider et al., 2019). Mindfulness has also been associated with improved positive affective responses to MVPA, leading to increased enjoyment and satisfaction and lower perceived exertion (Hardy and Rejeski, 1989) which have been associated with increased physical activity uptake and adherence (Williams et al., 2008; Tsafou et al., 2016). Changes in psychological factors such as habit, intention, motivation, self-efficacy and tolerance of discomfort are also associated with increases in mindfulness-related facets including attentional awareness, acceptance, and openness, suggesting these could, in part, contribute to both short-term increases in MVPA and readiness for longer-term behavior change (Schneider et al., 2019). In summary, existing literature has hypothesized many different factors that could explain the effects of mindfulness on MVPA including enhanced self-regulation abilities (e.g., attentional focus, behavioral flexibility, emotional relation) (Schneider et al., 2019), increased acceptance of uncomfortable thoughts and sensations (Arch and Craske, 2006), and changes in the stress response [i.e., changes in the hypothalamic–pituitary–adrenal (HPA) axis and autonomic nervous system] (Gamaiunova et al., 2022) that occur as an outcome of mindfulness practice.

Most studies examining the relation between MVPA and mindfulness targeted the two behaviors separately and demonstrated both increased mindfulness and increased physical activity adherence (Blair Kennedy and Resnick, 2015; Edwards and Loprinzi, 2018). To the best of our knowledge, only two studies have examined the effects of MVPA coupled with mindfulness training *during* an acute bout of activity [i.e., single period of activity (Basso and Suzuki, 2017)]. The first study used a within-subject design, and found listening to a mindfulness training video while engaging in an acute 10-min bout of moderate treadmill walking resulted in more positive affective valence, mindfulness and lower ratings of perceived exertion compared to walking alone (Cox et al., 2018). The second study was a between-groups randomized controlled trial in which participants randomized to 30-min of moderate intensity treadmill exercise while listening to a mindfulness recording for 7 days had higher self-reported MVPA and accelerometer MVPA as compared to the heart rate monitor only control group, and the combined activity and mindfulness intervention was feasible and acceptable (Sala et al., 2021). Although promising, these studies did not investigate objective exertion levels using physiological measures [i.e., blood pressure (BP), heart rate (HR)] to determine whether mindfulness training during physical activity alters the physiological response to exercise.

Thus, the purpose of this study was to examine the effects of an acute bout of MVPA (PA), mindfulness training (MIND), and combined MVPA and mindfulness training (PAMIND) on physiological and affective parameters. We hypothesized the MIND condition would result in the lowest physiological measure values and highest affective scores as compared to the other two conditions (PA and PAMIND). Further, we hypothesized that the PAMIND condition would result in lower perceived exertion ratings, HR response, and systolic blood pressure, and increased feeling ratings as compared to the PA condition.

## 2. Materials and methods

### 2.1. Study design

This study utilized a within-subjects, counter-balanced design. All participants were randomized 1:1 in a counterbalanced order to complete all three conditions: mindfulness only (MIND), physical activity only (PA), and physical activity while listening to a mindfulness audio recording (PAMIND). The within-subjects design allows for increased statistical power with a lower sample size, and controls for individual participant variables. Counterbalancing (i.e., randomizing the order in which a participant completes the study conditions) improves internal validity by controlling for sequence (e.g., practice) and order effects that may occur when participants repeatedly complete study conditions (Corriero, 2017).

### 2.2. Study procedures

Participants were recruited through self-referral using the Northwestern University Department of Psychology’s list of Paid Research Studies. Information about the present study was e-mailed to all individuals subscribed to the Paid Participant Registry. Individuals who were interested in participating were instructed to contact the study team via e-mail or phone after which they were emailed a link to the online screening survey to determine eligibility. Inclusion criteria were: (a) age 18–65 years old, (b) able to read, write, and speak in English, (c) able and willing to attend three in-person study visits, (d) access to a computer with Internet, (e) no self-reported respiratory, joint, or cardiovascular problems precluding physical activity, and (f) not pregnant or planning to become pregnant during the duration of the study. Eligible and interested participants completed an online informed consent. Participants were required to pass the Physical Activity Readiness Questionnaire (PAR-Q) (Adams, 1999) or obtain physician consent to participate. Within 48 h of passing screening, potential participants were emailed a copy of the study informed consent and a study overview document. Between 24 h to 5 business days after this email was sent, participants were called by study staff to confirm interest and eligibility. During this call, participants had the opportunity to ask questions about the study and informed consent. All eligible and interested individuals were emailed a secure, individualized link to complete the online informed consent. After informed consent completion, participants were emailed an individualized, secure link to the baseline assessment questionnaires. Upon completion of the baseline questionnaire, participants were randomized to the order of conditions and scheduled for their three study visits. All study procedures were approved by the institutional review board.

All study procedures occurred during three separate, individual in-person lab study visits separated by at least 48 h to ensure adequate recovery from physical activity. All participants were instructed to refrain from physical activity and caffeine consumption 6 h prior to each study visit to mitigate outside influence on HR or BP responses. Participants were fitted with a Polar H10 HR monitor (Polar Electro Oy, Kempele, Finland) (Gilgen-Ammann et al., 2019). Participants remained seated for a 5-min resting period during which measures of pre-condition Rating of Perceived Exertion (RPE) (Williams, 2017) and Feeling Scale (FS) (Hardy and Rejeski, 1989) were collected. At the end of the 5-min resting period, resting HR (two HR measurements from the Polar H10 monitor were recorded 1 min apart) and BP were measured.

**Figure 1:**
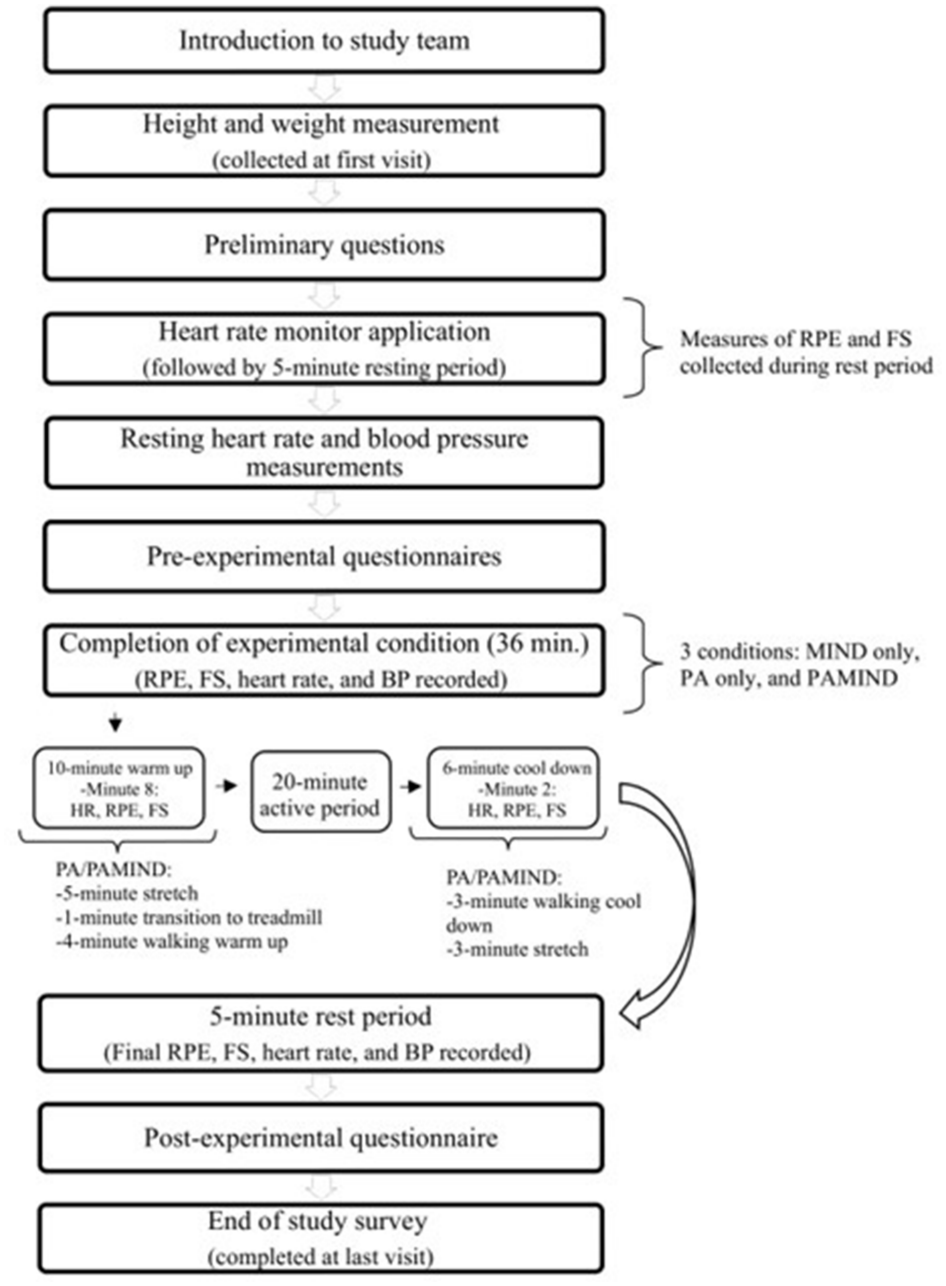
Participant flow through the study.

Following the resting period, pre-condition questionnaires were completed and followed by the 36-min experimental condition (i.e., MIND only, PA only, or PAMIND) to which they were assigned for that visit. RPE, FS, HR, and BP measurements were recorded once during the warm-up period and once during the cool down period. In addition, RPE, FS, and HR were recorded every minute during the 20-min active period, and BP was measured manually and recorded every 4 min. To prevent potential confounding of social interaction between the participant and study staff, talking was kept to a minimum at every visit. Upon completion of each experimental condition, participants engaged in seated rest for 5 min during which post-condition RPE, FS, HR and BP were measured. Finally, participants completed the post-condition questionnaires. Study visit procedures are demonstrated in Figure 1.

#### 2.2.1. MIND condition

Participants listened to a pre-recorded, 36-min, guided mindfulness meditation. The guided meditation instructed participants to lie on their back on a padded exam table, with the option to close their eyes at various points during the recording. The meditation emphasized bringing awareness to bodily sensations. The recording was structured as follows: (a) 10-min “warm-up,” (b) 20-min “active” mindfulness meditation, and (c) 6-min “cool down” period to match the PA and PAMIND protocols.

#### 2.2.2. PA condition

Prior to starting the condition, participants were familiarized with the treadmill controls and how to properly walk on a treadmill. The PA condition consisted of a: (a) 10-min warm-up (5 min of full body stretching and 5 min of easy walking), (b) 20-min brisk walk, and (c) 6-min cool down (3 min of light walking and 3 min of stretching). During the 20-min brisk walk, participants selected a speed and incline with the assistance of study staff to reach a moderate to vigorous intensity. The speed of the treadmill never increased to a point that required participants to jog. Rather, the incline was increased so that participants could maintain a walking pace and reach a moderate to vigorous intensity level of activity defined as 65%–85% of their maximum HR calculated by the Karvonen formula which takes into account resting heart rate and uses heart rate reserve (220-age) to estimate maximum HR (Karvonen et al., 1957). Study staff monitored participants’ heart rate in real-time to confirm participants were working at this moderate to vigorous intensity throughout the duration of the experimental condition.

#### 2.2.3. PAMIND condition

During this condition, participants walked on the treadmill following the exact same protocol as described above except they listened to a pre-recorded, 36-min, “Walking with Awareness” guided walking meditation during the warm-up, 20-min brisk walk, and the cool-down. Speed and incline were kept the same for each participant for the PA only and PAMIND condition to ensure consistency in intensity across conditions. A description of how this condition was developed is described below:

##### 2.2.3.1. Development of the PAMIND condition

The “walking with awareness” PAMIND component used in this study was led by co-author DV, who has significant experience teaching and conducting research in mindfulness and developing mindfulness-based clinical research interventions. Prior to writing and recording the script, several study team members participated in an audio recorded “think aloud” walking exercise on the treadmill, where they were asked to verbalize out loud everything they were noticing, observing, and experiencing across the five senses, thoughts, and emotions. This was done to gather rich, descriptive details of their walking experience that could be integrated into the recording and to pace the delivery of these details in line with the timeframe in which they were mentioned. This pre-testing activity was implemented by co-author WW.

During the 2-minute pre-walk stretch, participants made statements about noticing the environment they were in (e.g., cold floor, quietness, others watching them) while they stretched. They also commented on noticing muscle sensations of relaxation or tightness in the body. During the three-minute warm up on the treadmill, common observations included how this exercise differed from their usual workout routines (e.g., walking on a treadmill with no music while others watch). They also took notice of how their bodies felt as they warmed up, especially their legs. Another observation included how they focused on time due to no other distractions during the walk.

During the 20-min walk, observations were noted at each minute, which included:

- *Minutes 1–5*: Feeling bored without music and how their mind wandered to future activities; noticing the speed/sounds of the treadmill and breathing rate increasing following the warmup. Feeling their bodies awakening as they walked; beginning to think about their hunger and lunch plans; searching for something to look at in the room to distract themselves while they walked; being curious about the buttons on the treadmill; noticing the sounds of the treadmill while they scuffed their feet; checking in on their bodies including elevated heart rates, and unevenness of their gait.
- *Minutes 5–10*: Noticing skin warmth and being on the verge of breaking a sweat in their hands and other parts of their body; noticing other bodily sensations, such as their breathing increasing, goosebumps, or feeling their bodies loosening up; more comments about boredom and noticing the feeling of their arms swinging back and forth.
- *Minutes 10–15*: Describing tightness in legs; comments about wishing for a distraction such as music, and awkwardness of others in the room; noticing the pace they were walking, the body continuing to warm up, and the mind beginning to wander and think about what they planned to do later in the day; noting they would rather be outside than walking on a treadmill inside, as a poster inside of the room showed a picture of nature; one person reported feeling their body finally receiving the message that they were exercising and felt his body change not only movement-wise, but also temperature-wise; observations about physical state including dry mouth, muscles feelings more “awake” and feeling as though they were in a better mood.
- *Minutes 15–20*: Remarks of boredom and feeling uncomfortable making observations out loud while exercising; noticing breathing becoming heavier and how this exercise is atypical because they do not normally check in with how their bodies feel while working out, as they typically have external distractions such as listening to music/wearing a heart rate monitor; noticing breathing patterns and feelings in muscles that were working; feelings of breathing becoming heavier in comparison than the beginning of the exercise; noting the need to breathe through the mouth instead of the nose; realizing the walk is almost over and beginning to think about tasks they need to get done in the near future; noting it was mentally challenging to walk for such a long period of time on a treadmill without a change of scenery as exercising outside is motivating; remarking about being at peak physical exertion since the start of the exercise.

During the three-minute cool down on the treadmill, participants reported feeling that the pace was a drastic change from the speed they were previously walking at, as the cool down felt significantly slower. They noticed their heart rate and breathing slow down, their warmed-up bodies, and the feeling in the legs as the treadmill slowed. One person reported feeling accomplished about finishing the walk, and they were looking forward to being done and having a break. During the 2-min post walk stretch, participants reflected on enjoying the stretch and cool down and noticed how they felt looser now than at the start. They commented on the feeling of their bodies relaxing, and a comment was made about feeling more awake (mentally and physically).

Using descriptive statements from the “think aloud” exercises, a guided “walking with awareness” script was written that would lead participants through an 8-min warm up of stretching and warm up walking, 20-min of active treadmill walking, and 8-min of cool down (cool down walking and post-walk stretching). Photos of the intervention environment were taken to be able to visually describe the setting, which included the color of the walls, descriptions of photos and paintings, furniture, and room fixtures. A purposeful attempt was made to write and record these awareness-eliciting statements in a normal pace and vocal tone, so as not to induce a relaxation response, but rather seamlessly invite participants to notice and observe their multisensory experiences, thoughts, and feelings during the walking exercise. The final script was reviewed by the study team, and an audio recording was created by DV. Please see Appendix for the PAMIND script.

### 2.3. Measures

Table 1 details the measurement schedule for data collected during the study visits.

**Table 1:** Measurement schedule.

	Pre-condition	Post-condition	Warm-up	Cool-down	Every minute of condition	Every 4 min of condition
Heart rate	X*	X*	X	X	X	
Blood pressure	X	X	X	X		X
Feeling scale (FS)	X	X	X	X	X	
Rating of perceived exertion (RPE)	X	X	X	X	X	
State mindfulness scale (SMS)	X	X				
State trait anxiety inventory-state (STAI-S)	X	X				
Self-efficacy for walking duration (SEWD)	X	X				
Overall RPE		X				
Overall FS		X				
Overall enjoyment		X				

Values with * indicate a measure was recorded twice at a given time point.

#### 2.3.1. Physiological measures

##### 2.3.1.1. Height and weight

These measures were assessed during the first study visit prior to the start of the condition. Height and weight were measured objectively using a stadiometer for height (rounded to nearest 0.5 cm) and a body scale for weight (rounded to nearest 0.1 kg).

##### 2.3.1.2. Heart rate

HR was measured using a Polar H10 HR monitor (Polar Electro Oy, Kempele, Finland) (Gilgen-Ammann et al., 2019). Participants wore the Polar strap around the chest under their shirt, against their skin. The monitor was secured at the base of the sternum, just below the chest muscles, at the center of the chest. HR was measured twice during the pre-condition rest period (averaged to calculate pre-condition HR value), once during the walking phase of the warm-up, during each minute of the experimental condition, during the final minute of the cool-down, and twice during the post-condition rest period with the first reading occurring immediately following the cool down and the second reading 1 min later. HR measured via polar HR monitors worn around the chest is highly reliable and valid compared to electrocardiography (ECG) during rest and exercise (Plews et al., 2017; Gilgen-Ammann et al., 2019). Average and peak HR were calculated from the 20 readings taken during the 20-min active period during each condition.

##### 2.3.1.3. Blood pressure

BP was measured by staff manually with a sphygmomanometer and stethoscope once at rest pre-condition, once during the warm-up and cool down periods at the same time as HR, and once immediately post cool down at the same time as HR. It was also measured every 4 min during the experimental condition. BP was measured following standard procedures (Beevers et al., 2001) for rest and standard procedures for obtaining an exercising BP (Myers et al., 2009). The average BP from the 6 readings taken during the 20-min active portion of each condition, and peak BP were calculated.

#### 2.3.2. Psychological measures

##### 2.3.2.1. Pre-, during and post-condition measures

These measures were taken immediately pre-condition, once during the warm-up, at each minute during the active condition, once during the cool down and 5 min, and after the cool down period ended (i.e., post-condition). Average ratings were calculated for each measure using the 20 readings taken during each 20-min active portion of each condition. Peak ratings were the highest value reported (RPE, FS) or collected (HR, BP) at any of the 20 during-test readings.

**Feeling scale (FS).** The FS measures affective valence at the present moment with one item “How do you feel right now?” on an 11-point scale from −5 (Very bad) to +5 (Very good) (Hardy and Rejeski, 1989). The FS is a valid and reliable measure of affective valance and is shown to correlate with other measures of affect (Hall et al., 2002). It has been widely used to measure affective valence in physical activity studies (Ekkekakis, 2003).

**Rating of Perceived Exertion (RPE).** RPE measures subjective physical activity intensity. The Borg RPE scale consists of numerical values from 6–20 to represent the effort at which an individual perceives they are exerting themselves from rest (6) to extreme exertion (20). Participants are asked “How hard does it feel like you are working”? They are instructed that their rating should reflect their total amount of exertion and fatigue and to not focus on any one factor such as leg fatigue or shortness of breath. The design of the RPE scale is to mirror that of the average human HR range: 60 bpm to 200 bpm (Williams, 2017). RPE has shown to be a valid and reliable estimation measure of perceived exertion during rest and exercise in adults (Chen et al., 2002).

##### 2.3.2.2. Pre- and post-condition measures

**Mindfulness.** The 21-item State Mindfulness Scale (SMS) was used to retrospectively measure various qualities of mindful awareness during each experimental condition. Participants indicated how well each statement describes their recent experience (e.g., awareness of emotions, physical sensations, thoughts, and surroundings) on a scale from 1 (not at all) to 5 (very well). The SMS was used because it has two subscales, (1) mindfulness of bodily sensations (6 items) and (2) mindfulness of mental events (15 items) that can provide insight into components of mindfulness that are pertinent to physical activity. Items are summed and averaged to obtain subscale scores ranging from 1 to 5, with higher scores indicating higher levels of state mindfulness. This scale is valid, stable across time and context and is sensitive to change (Tanay and Bernstein, 2013) and has been used in prior studies examining the effect of incorporating mindfulness into acute bouts of physical activity (Cox et al., 2018).

**State anxiety.** The 20-item State Trait Anxiety Inventory-State (STAI-S), a valid and reliable measure of current anxiety (Metzger, 1976; Spielberger, 1989), assessed how participants felt right now at this moment based on various statements (i.e., “I feel calm” or “I feel nervous and restless”) on a scale from 1 (Not at all) to 4 (Very much so). Item values were summed to obtain total STAI-S scores ranging from 20 to 80, with higher scores indicating higher levels of state anxiety.

**Self-efficacy.** The Self-Efficacy of Walking for Duration (SEW-D) scale is a 10-item scale that asks participants to rate how confident they feel that they could complete incremental 5-min intervals (5–50 min). Participants rate confidence levels to execute the behavior on a 100-point percentage scale comprised of 10-point increments, ranging from 0% (not at all confident) to 100% (highly confident). Total self-efficacy is calculated by summing each item response and dividing by the total number of items in the scale ranging from 0 to 100. The SEWD is a valid and reliable measure of walking self-efficacy for duration (McAuley et al., 2000).

#### 2.3.3. Post-condition overall measures

Participants were asked to indicate their overall RPE, FS, and enjoyment of the specific condition completed at the conclusion of each condition by answering a one-item question about each construct. The RPE scale and FS measure question phrasing was edited to instruct participants to provide overall estimation of RPE and FS from the completed session, rather than measuring these constructs within the current moment. For overall enjoyment, participants were asked to rate their overall enjoyment of the completed condition on a scale from 1 (I enjoy it) to 7 (I hate it).

### 2.4. Statistical analysis

An *a priori* power analysis was conducted for sample size estimation. The required sample size to achieve 80% power for detecting a small to medium effect size (η^2^ ≥ 0.04) from pre- to post-session between the three conditions was *N* = 24. Descriptive analyses were conducted using SPSS Version 25 (IBM Corp, Armonk, NY, United States). Means and standard deviations of each variable were calculated for each condition. A one-factor repeated-measures ANOVA was used to examine the differences by condition on average and peak HR, BP, FS and RPE. A three (condition) by two (time) within-subjects repeated measures ANOVA was used to examine the effects of each of the three conditions on differences within and across conditions for the pre- and post-condition measures of HR, BP, FS, RPE, SMS, STAI-S and self-efficacy and post-condition only measures of RPE, FS and enjoyment. Time and condition were included as within-subjects factors to allow each subject to serve as their own “control.” Bonferroni corrections were used to correct for the multiple tests conducted.

## 3. Results

### 3.1. Participants

All three conditions were completed by 29 individuals who are included in analyses. Participant demographic characteristics are presented in Table 2. Participants were on average 28.59 years old, 55% underrepresented race/ethnicity, with an average BMI of 26.67. Most participants were female (65.5%), had a college degree (72.4%), and rated their overall health status as excellent/very good (58.6%). Participant recruitment and retention is detailed elsewhere (Torre et al., 2023). Briefly, screening surveys were sent to 180 individuals and 60% (*n* = 108) were eligible to receive a recruitment call. In total, 59 recruitment calls were attempted and 72.9% (*n* = 43) were completed, resulting in 39 participants signing the informed consent form and being randomized. Of those randomized, 79.5% (*n* = 31) completed their first study visit, and 93.5% (*n* = 29) of these participants were retained through all three study visits.

**Table 2:** Participant characteristics [*n* (%) for categorial variables and mean (SD) for continuous variables].

Full sample (*N* = 29)	
Age (*M*[SD])	28.59 [9.0]
BMI (*M*[SD])	26.67 [6.1]
Exercise self-efficacy (*M*[SD])	8.59 [2.53]
Gender	
Female	19 (65.5)
Male	10 (34.5)
Race	
White	13 (44.8)
African American	6 (20.7)
Asian/Pacific Islander	8 (27.6)
Other	2 (6.8)
Hispanic or Latino	4 (13.8)
≥College degree	21 (72.4)
Working at least part-time	24 (82.8)
Annual Household Income	
< $60,001	18 (62.1)
Prefer not to answer	3 (10.3)
Overall Health Status	
Excellent /very good	17 (58.6)
Good	11 (37.9)
Fair/poor	1 (3.4)

### 3.2. Physiological measures

All within-subject differences between average and peak physiological measures collected during the experimental conditions (i.e., BP, HR) are demonstrated in Table 3. All within-subject differences between pre- and post-condition measures are shown in Table 4.

**Table 3:** Within-subject differences between average and peak physiological and affective measures by experimental condition.

Measure	Condition			*p*-value
	MIND *M*(SD) (a)	PA *M*(SD) (b)	PA/MIND *M*(SD) (c)	
Systolic blood pressure				
Average	113.0 (10.3)^b,c^	120.4 (10.7)^a^	120.9 (11.3)^a^	<0.001
Peak	115.1 (10.7)^b,c^	124.9 (11.6)^a^	127.5 (12.2)^a^	<0.001
Diastolic blood pressure				
Average	70.9 (11.2)	67.2 (9.0)	69.6 (9.3)	0.07
Peak	72.6 (11.7)	69.1 (9.3)	72.3 (13.2)	0.15
Heart rate				
Average	64.5 (8.7)^b,c^	132.9 (10.5)^a^	128.9 (16.9)^a^	<0.001
Peak	69.7 (9.7)^b,c^	145.7 (10.1)^a^	142.4 (15.3)^a^	<0.001
Rating of perceived exertion				
Average	6.2 (0.4)^b,c^	12.4 (2.3)^a,c^	11.2 (2.0)^a,b^	<0.001
Peak	6.4 (0.8)^b,c^	13.6 (2.6)^a^	13.3 (2.2)^a^	<0.001
Overall	6.4 (0.7) ^b,c^	12.2 (2.4)^a^	12.1 (2.3)^a^	<0.001
Feeling scale				
Average	3.6 (1.0)^b,c^	2.3 (1.4)^a^	2.3 (1.3)^a^	<0.001
Peak	4.1 (0.99)^b,c^	3.1 (1.3)^a^	3.1 (1.1)^a^	<0.001
Overall	3.9 (1.3)^b,c^	1.7 (1.9)^a^	2.0 (2.4)^a^	0.008
Enjoyment				
Overall	2.07 (1.2)^b,c^	2.9 (1.4)^a^	2.9 (1.7)^a^	0.009

^a,b,c^ Indicate significant between condition differences at *p* < 0.05 where a = MIND, b = PA, and c = PAMIND.

**Table 4:** Average and difference of pre/post condition scores.

Measure	MIND *M* (SD)		PA *M* (SD)		PAMIND *M* (SD)		*p*-value (effect size, η^2^)		
	Pre	Post	Pre	Post	Pre	Post	Condition	Time	Condition × time
Systolic BP	115.86* (2.14)	112.57* (2.03)	113.71 (10.83)	112.68 (11.20)	111.43 (11.35)	111.86 (12.41)	0.31 (0.04)	0.11 (0.09)	0.12 (0.08)
Diastolic BP	69.86 (10.98)	71.43 (10.94)	67.43 (9.06)	68.82 (9.09)	69.96 (9.23)	70.71 (9.21)	0.25 (0.50)	**0.02 (0.18)**	0.77 (0.01)
HR	78.30* (2.34)	74.07* (1.87)	79.75* (2.64)	89.45* (2.67)	81.27* (2.76)	87.31* (2.55)	**<0.001 (0.39)**	**<0.001 (0.47)**	**<0.001 (0.53)**
Rating of perceived exertion	6.07 (0.05)	6.10 (0.06)	6.24 (0.15)	6.31 (0.10)	6.28 (0.16)	6.38 (0.16)	**0.04 (0.11)**	0.39 (0.03)	0.80 (0.01)
Feeling scale	2.90* (0.25)	3.62* (0.21)	2.79* (0.30)	3.28* (0.25)	2.69* (0.26)	3.14* (0.26)	0.19 (0.06)	**<0.001 (0.38)**	0.54 (0.02)
Mindfulness (Body)	2.20* (0.91)	3.57* (0.82)	2.12* (1.03)	3.19* (0.99)	2.02* (0.92)	3.41* (1.20)	0.24 (0.05)	**<0.001 (0.71)**	0.39 (0.04)
Mindfulness (mental event)	2.80* (0.86)	3.48* (0.72)	2.72 (0.92)	2.93 (0.85)	2.71* (0.84)	3.21* (0.96)	0.09 (0.09)	**0.001 (0.37)**	**0.03 (0.13)**
State anxiety	35.37* (0.23)	31.70* (8.91)	33.22 (9.23)	33.33 (9.09)	34.48 (8.61)	34.37 (11.35)	0.53 (0.02)	0.25 (0.05)	**0.01 (0.15)**
Self-efficacy for walking	9.49 (1.74)	9.78 (1.66)	9.47 (1.64)	9.71 (1.47)	9.33 (2.00)	9.74 (1.47)	0.91 (0.00)	**0.04 (0.15)**	0.86 (0.01)

Bold indicates *p* ≤ 0.05. * indicates a significant difference between pre- and post-condition.

#### 3.2.1. Blood pressure

Compared to the MIND condition, average and peak SBP were significantly higher during the PA and PAMIND conditions (*p’*s ≤ 0.001), independently. There were no differences between the PA and PAMIND conditions for average or peak SBP. Average and peak DBP did not differ by condition. There were no significant main effects of condition or time or time by condition interaction effects for SBP. For DBP, there was a significant effect of time [*F*(1, 27) = 5.89, *p =* 0.02, partial η^2^ = 0.18] such that diastolic blood pressure increased across all conditions from pre-to post-condition.

#### 3.2.2. Heart rate

Average and peak HR were significantly higher during the PA and PAMIND conditions, independently, compared to the MIND condition (*p’*s ≤ 0.001). There were no differences between the PA and PAMIND conditions for average or peak HR. There was a statistically significant main effect of time [*F*(1, 27) = 23.90, *p* < 0.001, partial η^2^ = 0.47] and condition [*F*(2, 26) = 17.57, *p* < 0.001, partial η^2^ = 0.39], and a statistically significant time by condition interaction [*F*(2, 26) =30.86, *p* < 0.001, partial η^2^ = 0.53] for HR (*p*’s < 0.001). In the PA and PAMIND conditions, HR significantly increased pre- to post-condition (*p* < 0.001) while HR significantly decreased pre- to post- for MIND (*p* < 0.01).

### 3.3. Psychological measures

All within-subject differences between average and peak psychological measures collected during the experimental conditions (i.e., RPE, FS) are demonstrated in Table 3. All within-subject differences between pre- and post-condition measures are shown in Table 4.

#### 3.3.1. Rating of perceived exertion

There was a statistically significant main effect of condition [*F*(2, 26) = 3.32, *p* = 0.04, partial η^2^ = 0.11] on RPE. Average and peak RPE were significantly higher during the PA and PAMIND conditions, independently, compared to the MIND condition (*p*’s < 0.001). Average RPE was significantly lower during PAMIND compared to PA (*p* < 0.001). There were no differences between the PA and PAMIND conditions for peak RPE.

#### 3.3.2. Feeling scale

Average and peak FS were significantly lower during both the PA and PAMIND conditions, independently, compared to the MIND condition (*p*’s < 0.001). There were no differences between the PA and PAMIND conditions on FS measures. There was a statistically significant main effect of time [*F*(1, 27) = 16.78, *p* < 0.001, partial η^2^ = 0.38], demonstrating FS scores increased significantly pre- to post-condition across all conditions, demonstrating more positive affective valence.

#### 3.3.3. Mindfulness

There was a significant increase in mindfulness of bodily sensations [*F*(1, 27) = 64.74, *p* < 0.001, partial η^2^ = 0.71] and mindfulness of mental events [*F*(1, 27) = 15.18, *p* = 0.001, partial η ^2^ = 0.37] from pre- to post-condition across all conditions. There was also a statistically significant time by condition interaction effect for mindfulness of mental events [*F*(2, 26) = 3.92, *p* = 0.03, partial η^2^ = 0.13]. Such that mindfulness for mental events increased more in the PAMIND condition than the PA condition.

#### 3.3.4. State anxiety

There was a statistically significant time by condition interaction effect for state anxiety [*F*(2, 26) = 4.73, *p* = 0.01, partial η^2^ = 0.15] such that anxiety decreased more for the MIND condition than the PA or PAMIND condition.

#### 3.3.5. Self-efficacy

There was a statistically significant effect of time on self-efficacy for walking [*F*(1, 27) = 4.71, *p* = 0.04, partial η = 0.15].The main effect of condition and the time by condition interaction were not significant.

### 3.4. Post-condition overall measures

Overall post-condition ratings of session RPE, FS and enjoyment were significantly different between MIND only and PA only conditions and MIND only and PAMIND conditions. Overall ratings for RPE (*p* < 0.001) and enjoyment (*p* < 0.05) were significantly lower for the MIND only condition and overall FS ratings (*p* < 0.05) were significantly higher for MIND only condition as compared to the PA only and the PAMIND conditions. This indicates that, on average, participants reported significantly lower levels of perceived exertion, more positive affective valence, and higher ratings of enjoyment, overall, during the MIND condition as compared to PA and PAMIND conditions. Post-condition overall ratings of RPE, FS, and enjoyment were not significantly different between PA only and PAMIND conditions.

## 4. Discussion

The primary goal of this study was to examine the effects of guided mindfulness meditation during MVPA on physiological and psychological parameters by comparing three conditions: mindfulness only, physical activity only, and combined physical activity and mindfulness. The PAMIND condition resulted in lower perceived exertion ratings (but not physiological parameters or feeling scale ratings) compared to the PA condition, which had equivalent intensity and duration. Additionally, mindfulness of mental events increased pre- to post-condition for the MIND and PAMIND conditions, but not in the PA condition. Overall, these findings suggest mindfulness during MVPA could provide benefits via effects on RPE and mindfulness of mental events.

### 4.1. Theoretical contributions

We observed no significant differences between the PA only condition and the PAMIND condition in HR or BP. Although mindfulness training has been shown to have beneficial effects on physiological markers of stress, including HR and BP (Ditto et al., 2006; Kingston et al., 2007; Delgado et al., 2010; Pascoe et al., 2017; Gamaiunova et al., 2022) the pairing of guided mindfulness with an acute bout of MVPA did not influence immediate physiological responses. It is likely that the acute effects of MVPA for stimulating HR and BP outweighed the acute blunting effect of mindfulness on these physiological responses. Indeed, reduced physiological response as a function of engaging in mindfulness during MVPA may not be expected or desirable. Rather, it will be important for future studies to explore whether there are long-term effects of practicing mindfulness while engaging in MVPA on BP and HR.

Interestingly, even though physiological parameters did not differ, average RPE did differ when participants completed the same PA with and without accompanying mindfulness. In that, participants perceived the exercise to be less strenuous, on average, when mindfulness was combined with PA than when they did PA alone. Existing literature states that perceived exertion is an outcome of both psychological and physiological components (Rejeski, 1981). Our findings support that, in a controlled setting, psychological intervention (i.e., mindfulness) can influence perceived exertion when the physiological response to activity is unchanged. Practicing mindfulness simultaneously with MVPA may increase distress tolerance during PA and attenuate how hard one feels that they are working. This could be a result of individuals more accurately interpreting their physiological response to MVPA, resulting in lower perceptions of exertion (Cox et al., 2018; Meggs and Chen, 2021). Notably, it is possible that the underlying factors explaining lower RPE ratings might be distraction offered by listening to the guided audio tape while walking on the treadmill during the PAMIND condition. Prior studies support that attentional distraction and sensory deprivation while exercising (e.g., listening to music) is associated with lower RPE as compared to control groups (Boutcher and Trenske, 1990; Yamashita et al., 2006; Gillman and Bryan, 2019). Therefore, future studies might consider controlling for distraction by comparing PAMIND to a condition in which participants listen to another type of audio (e.g., music, audiobook, podcast) while walking on a treadmill.

Our findings regarding psychological variables were mixed. The feeling scale, body and mental event mindfulness and self-efficacy all improved pre- to post-test regardless of condition suggesting all three conditions have positive acute effects on psychological outcomes. There was a significant time by condition interaction such that state anxiety decreased more for the MIND condition than the PA or PAMIND condition, although it was decreased across all conditions. Additionally, there was a significant time by condition interaction for mindfulness of mental events such that mindfulness of mental events increased more in the PAMIND condition than the PA condition. These findings suggest there could be differential acute effects of the three conditions on psychological outcomes favoring the MIND or PAMIND conditions.

Overall, our study addresses gaps in the literature by investigating the physiological and psychological outcomes of practicing mindfulness during an acute bout of MVPA. Our findings are similar to Cox and colleagues’ (Cox et al., 2018) study that combined listening to a mindfulness recording while walking on the treadmill regarding decreased RPE, and increased mindfulness but did not replicate results concerning positive affect or enjoyment. The discrepancy in results might be explained by the fact that Cox and colleagues recruited individuals who were, on average, younger and reported low intrinsic motivation to exercise. The present study did not include inclusion criteria related to exercise motivation or enjoyment, and therefore, might have enrolled participants who self-reported high baseline levels of exercise enjoyment and more positive affect associated with PA. Therefore, there could be a ceiling effect for affect and enjoyment, as participants in the present study might not have had much room for improvement in these measures.

### 4.2. Practical implications

Findings from this study have practical implications that may guide future development of MVPA promotion interventions or policy. Firstly, the finding that RPE significantly differed between the PA and PAMIND conditions without changes in physiologic responses to activity has potential implications for exercise initiation and maintenance. High perceived exertion can be a barrier to PA participation (Lind et al., 2009). The potential for mindfulness to reduce perceived exertion for the same intensity activity, even when physiological responses are the same, could make exercise more tolerable resulting in enhanced physical activity initiation and maintenance and increased engagement in a higher dose (duration, intensity, or frequency) of MVPA. Second, this study demonstrated a significantly greater increase in mindfulness of mental events in the PAMIND condition as compared to the PA only condition. Practically, given that mindful awareness facilitates enhanced emotion regulation (Roemer et al., 2015), promoting mindfulness during physical activity could enhance MVPA participation by reducing negative and increasing positive emotional responses. Ultimately, integrating mindfulness and MVPA has potential to improve adherence to MVPA guidelines resulting in improved health outcomes and reducing disease risk. Mindfulness is growing in global popularity (Misitzis, 2020), and increased evidence of its health benefits are emerging (e.g., stress reduction, improved mental health, lowered BP, etc.) (Donald et al., 2016; Hofmann and Gómez, 2017; Pascoe et al., 2017; Gamaiunova et al., 2022). Although there are no official public health guidelines for mindfulness practice, it is possible such guidelines will be developed as research continues to support mindfulness practice as a feasible, low cost, and efficacious health behavior. As guidelines and policies are developed, considering the potential effect of mindfulness on MVPA engagement offers potential for combined recommendations that could promote MVPA adherence and compound health benefits. Future studies are warranted to further examine the acute and long-term effects of combining physical activity and mindfulness and potential biopsychosocial mechanisms by which mindfulness could enhance MVPA participation.

### 4.3. Limitations

This study is not without limitations. First, this research was conducted with young, healthy adults who already engage in various levels of physical activity. Future studies should examine whether these results are replicable in other populations including older adults, individuals with chronic conditions, varying activity levels, and an aversion to, or difficulty with, increased exercise. Next, this study only examined one type of physical activity that is likely familiar to participants (treadmill walking) for one 20-min session in a controlled, laboratory setting. Future work should explore whether findings differ based on different activity characteristics, including, but not limited to type (e.g., bike, running), intensity, duration, and familiarity. Additionally, it is important to examine these relationships in various contexts (e.g., indoor v. outdoors) and in participants’ natural environments to better understand whether these factors differentially influence how mindfulness may influence physiological and psychological parameters and MVPA participation. More research is also needed to examine whether decreased RPE during a single bout of mindful PA increases motivation or engagement in future MVPA, or what dose of intervention is necessary to increase overall MVPA engagement and adherence. Lastly, although objective data was included in this study (i.e., HR, BP), many variables were collected via self-report measures (i.e., RPE, FS, enjoyment, mindfulness, state anxiety, self-efficacy) and reported in real-time to study staff. These data collection procedures can potentially create bias in self-reported data. Incorporating additional objective measurements (e.g., heart rate variability, lactate levels, VO2 max) in future studies may strengthen reliability and provide additional insight into potential differences in physiological responses during exercise.

## 5. Conclusion

Despite these limitations, this is the first study to test the acute effects of combined mindfulness and PA training on psychological and physiological outcomes. Additional strengths include the within-person randomized-controlled trial design and the inclusion of both mindfulness-only and physical activity-only conditions. Our findings are promising in terms of both the promotion of physical activity and the efficacy of combining mindfulness with acute bouts of MVPA. Future research is needed to determine if our findings can be replicated to examine the underlying factors by which combining mindfulness and PA may decrease ratings of perceived exertion, even without differences in physiological responses compared to PA only. Ultimately, these findings provide initial support to justify further exploration of the effects of combining mindfulness and MVPA as a strategy to promote PA. Given that limited equipment is required, and mindfulness can be done anywhere, mindfulness combined with PA may represent a low-cost, highly disseminable physical activity promotion strategy that could substantially impact public health.

## Data availability statement

The raw data supporting the conclusions of this article will be made available by the authors, without undue reservation.

## Ethics statement

The studies involving humans were approved by Northwestern University Institutional Review Board (IRB). The studies were conducted in accordance with the local legislation and institutional requirements. The participants provided their written informed consent to participate in this study.

## Author contributions

PS: Writing – original draft, Writing – review & editing, Formal analysis, Investigation, Project administration. LA-G: Data curation, Formal analysis, Writing – original draft, Writing – review & editing. ET: Investigation, Writing – review & editing, Project administration. WW: Formal analysis, Writing – review & editing, Data curation, Methodology, Project administration. KM: Writing – review & editing. JS: Writing – review & editing. JR: Writing – review & editing. DV: Conceptualization, Funding acquisition, Methodology, Supervision, Writing – review & editing. SP: Conceptualization, Formal analysis, Funding acquisition, Methodology, Project administration, Supervision, Writing – review & editing, Resources, Validation, Writing – original draft.
