# Supplementary material for: Effects of mindful physical activity on perceived exercise exertion and other physiological and psychological responses: results from a within-subjects, counter-balanced study
Source: Front Psychol. 2023 Nov 10;14:1285315. doi: 10.3389/fpsyg.2023.1285315 (PMC10667420; doi:10.3389/fpsyg.2023.1285315)
Supplement: Supplementary file 1 [file Data_Sheet_1.PDF]

PAMIND "Walking with Awareness" Practice

[INTRODUCTION & WARM UP STRETCH] – 2 Minutes

This is the "walking with awareness" practice.

During this practice we will begin with a few gentle stretches to warm up the body, followed by a period of walking on the treadmill, and then finishing with another brief cool down period.

Find an open area on the floor where you may comfortably sit down with your legs in an extended position in front of you.

Before you start stretching, let's begin by simply bringing your awareness to the room itself and seeing what you might notice here. Maybe it's the color of the paint on the walls or the lighting, maybe it's how the room is decorated, maybe it's the different pieces of furniture or equipment…whatever the eyes are viewing right now, see if it's possible to simply notice some of the different things that make up this room, not getting overly fixated on any one thing, but rather taking it all in.

As you find yourself sitting here, take a moment to just notice where your body is coming into contact with the floor, and what this is like. Maybe you're noticing the temperature of the floor, maybe how your posture is aligned, maybe you can feel or even hear your heart beat or some other sound coming from within your body. There might be different sounds you become aware of from the outside environment, or even a lack of sounds. Whatever your experience is right now, simply let yourself notice and observe it, almost as if it were for the very first time. This kind of noticing is called "beginner's mind", which can help us learn to experience things we're used to in a different, less automatic way.

Let's transition now to warming up the body with a few gentle stretches. As you stretch, see if you can notice some of the different sensations in your body, from tightness, to relaxation. Make sure that you only do what you feel comfortable with, trying to avoid pushing or overexerting yourself to the point of feeling pain or possibly hurting yourself. Remember, this is simply a gentle stretch to wake up the body before you start walking.

Let's begin.

Lying back on the floor, slowly bring your knees up to your chest and cross your arms around them to help hold the position. Make sure not to hold your breath, and to just allow your breathing to take place in a natural and normal manner. Hold this for around 20 seconds and see if you can just notice what this feels like in the body, from the stretch in your legs to your lower back. If it feels right, you might also let yourself gently rock from side to side.

[20 seconds]

Now let your legs slowly return back to the floor in the outright position, again noticing some of the different sensations in the body as you do this.

And again, bringing your legs back up to your chest to repeat this stretch, making sure to continue breathing in a normal and natural way, paying attention to whatever arises with curiosity.

[20 seconds]

Now let's move the body to an upright seated position with your legs extended straight out in front of you. Without bending your knees, see if you can flex your feet so that your toes are pointing toward you. And just hold that for a few seconds. What does this feel like in your legs? Where do you feel it? Let go of the flex, and again notice the different sensations between tension and relaxation when you do this. Repeat this pattern of toe flexing and releasing for next few seconds.

[10 seconds]

Now, slowly extending your arms and hands forward, without putting undue strain on your lower back, gently reach out toward your toes, while flexing your toes towards your hands. Maybe you can touch or hold them, or maybe that doesn't feel so comfortable. It doesn't really matter. What's most important is that you are leaning forward with your hands and arms extended in this outright position toward your toes. Just noticing where the stretch is, and how it feels. Instead of labeling this physical sensation as feeling "great" or possibly "difficult", see if you can instead simply experience this sensation as being "pleasant" or "unpleasant" or even "neutral."

Let's stay in this reaching position for a few more seconds. In addition to noticing physical sensations, you might also become aware of certain thoughts or emotions about what you're doing. Maybe you feel proud or happy with how flexible you are. Maybe you're thinking that you wished you were more flexible, or that you don't think you're very good at this. Whatever thoughts and emotions that might arise, see if you can practice beginner's mind of just noticing them all with openness and curiosity, not needing to change them in any way.

And releasing this stretch, bringing your hands and arms back to your sides.

When you're ready, go ahead and stand up, and for the next few moments stretch out any other parts of your body that need some more attention, maybe pushing against the wall to stretch out your calf muscles, rotating your arms in circular motions, twisting your torso from side to side or slowly moving your head and neck in a circular motion. Whatever needs more stretching go ahead and do that now.

[10-15 seconds]

[INTRODUCTION & WALKING WARM UP] – 3 minutes

We will now transition to the treadmill where you will start out at a slower pace to help your legs and body get used to walking in this way. Please let the research assistant know that you are ready to get onto the treadmill to begin this part. There will be silence for the next minute so that you can get set up with this.

[1 minute]

And as you walk here, right now, see if it's possible to bring your awareness to the full act of walking, with one foot in front of the other, noticing the natural rhythm, pace, and cadence, and that while the body knows exactly what to do, a lot of complex actions are involved in this simple act of walking, especially when walking on a surface that is moving like a treadmill, from the weight of each step lifting

up and stepping back down on the track, to the body's continual re-positioning, and use of other body parts, like the arms, to help maintain balance and stability, just see if you can take in an observe the entire body as a whole, walking right now in this moment.

[20 seconds]

Whenever we begin to pay full attention to something like this, whether it's noticing the experience of walking or observing our breath coming in and going out, at some point, it's inevitable for our attention to wander elsewhere. There might be outside sounds and distractions that take us away. We might start thinking about something that happened earlier in the day, or begin planning something that will occur in the future. Our attention may wander to different sensations, thoughts or emotions related to the experience itself, like "my body is starting to feel warmer" or "I'm glad I'm getting my steps in today" or "this is kind of boring", or "my mood is feeling better." As you continue, you might experience similar sensations, thoughts and emotions. See if you can notice this with curiosity when it occurs, and instead of indludging every distraction that takes you away, see if it's possible to anchor yourself back to the present moment of walking. You might even silently say to yourself, "Walking. This is walking" and come back to noticing the full experience of putting one foot over the other, from one moment to the next.

[TREADMILL WALK] – 20 minutes

We are now ready to begin the 20-minute period of treadmill walking at a slightly faster pace. Please let the research assistant know that you are ready for this. There will be silence for the next minute so that you can get set up.

Now that you're walking at a faster pace, similar to before, see if you can begin to just notice the experience of walking here on this treadmill. As you continue, your mind might wander off to thoughts about how you usually exercise with music and how this might be different. You might notice different sounds, like the sound coming from the treadmill motor or what your steps sound like as they move along. You might even begin to notice your breathing start to increase or the feeling of your body waking up with this movement. Whatever it is, let yourself notice it, try not to judge it as being good or bad, but rather using it as a cue to anchor your awareness and focus back to your body and the full experience of walking, right now and right here, from moment to moment.

[Silence for 4-5 minutes]

And checking back in to see where the mind may have wandered. Maybe you've started to become aware of increases in heart rate, possibly noticing skin beginning to feel warmer, maybe even breaking a sweat and feeling the muscles starting to loosen up. Wherever the mind has gone, remembering to simply notice that it has drifted away, and to gently bring your awareness back to the full experience of walking, right here in this present moment.

[Silence for 4-5 minutes]

At this point in this exercise, it is not uncommon for the mind to continue to wander, maybe wishing for a distraction such as music, maybe being aware of the pace or the body continuing to warm up, or maybe even thinking about what you planned to do later in the day. Maybe there are thoughts about wishing this exercise was taking place outside rather than here on this treadmill, or possibly starting to

notice different physical states, like a dry mouth, muscles feelings more ‘awake’, or breathing more heavily. Whatever the different observations are, similar to before, after you notice that your focus has drifted down stream, very gently and calmly, escort your attention back to this experience of walking.

[Silence for 4-5 minutes]

And once again checking in to see where the mind may have wandered. At this point in this exercise it is common to be aware that breathing and physical exertion have increased since the start of this walk, maybe there’s a realization that you are approaching the end of this exercise, which can trigger many different thoughts and emotions about what is next on your list after this, where you will be and what you will do. Instead of going there just yet, however, see if you can stay connected to this exercise with present moment awareness of the full experience of your body walking here on this treadmill, right now.

[Silence for 4 minutes]

You have now completed your 20-minutes of paced walking, so please let the research assistant know that you are ready to slow the pace down for a brief cool-down period. There will be silence for the next minute so that you can get set up.

[Cool Down Walk] 3 minutes

As you walk now at this slower pace, see if it’s possible to bring awareness to your body, and notice if this experience might be different than walking at a fast pace. Maybe it is, maybe it isn’t. Simple let yourself become aware of what the body feels like right now. What physical sensations are you aware of right now? As you cool down, continue to stay tuned into your body walking at a slower pace. See if you can stay with it, and when your attention drifts elsewhere, just recognize it and gently bring your awareness back.

[Silence]

You have now completed your cool down walking, so please let the research assistant know that you are ready to turn off the treadmill and return to the floor for some gentle stretches. When you have gotten off the treadmill, you can start by getting into a comfortable position lying down on your back. There will be silence for the next minute so that you can get set up.

[1 Minute]

[Cool Down Stretch] – 2 minutes

As you lie here on your back, just allow your body to gently rest on floor, bringing your full awareness to your legs, hips and waist, lower and upper back, shoulders, arms, hands, neck, and head. Your entire body. Allow yourself to take a few deep, relaxing breaths, slowly breathing in from the stomach, inhaling through your nostrils, and exhaling though the mouth, noticing the air as it enters the body with each

inhalation, and exits the body with each exhalation. Just being open and curious to any sensations that may arise. Maybe changes in temperature, sensations of lightness and floating, or possibly heaviness and sinking, noticing the muscles and how they feel, loose? Tight? Sore? Relaxed? An absence of sensation? Whatever the experience is, simply bring your awareness to it, and stay connected with it as long as you can. And when your attention is taken elsewhere, simply redirect it back to the sensations of the body.

[1 minute]

Congratulations, you have now completed this walking program. Please slowly make your way back to a standing position and let the research assistant know that you are done.
